# InCASEOf scoring system for distinction between pachychoroid-associated macular neovascularization and neovascular age-related macular degeneration in patients older than 50 years

**DOI:** 10.1038/s41598-022-06968-0

**Published:** 2022-02-21

**Authors:** Grazia M. Cozzupoli, Enrico Borrelli, Vittorio Capuano, Riccardo Sacconi, Polina Astroz, Marco Battista, Francesco Bandello, Eric Souied, Giuseppe Querques

**Affiliations:** 1 Department of Ophthalmology, Centre Hospitalier Intercommunal de Créteil, University Paris-Est Créteil, Créteil, France; 2 School of Medicine, Vita-Salute San Raffaele University, Milan, Italy; 3 Division of Head and Neck, Ophthalmology Unit, IRCCS San Raffaele Scientific Institute, Milan, Italy; 4 Department of Ophthalmology, IRCCS Ospedale San Raffaele, Vita-Salute University, Via Olgettina, 58, 20132 Milan, Italy

**Keywords:** Visual system, Retina, Retinal diseases, Macular degeneration

## Abstract

To develop a novel scoring system aiming at guiding the differential diagnosis between macular neovascularization secondary to pachychoroid disease (pMNV) and neovascular age-related macular degeneration (AMD) in patients aged 50 years and older. In this retrospective study performed at University Vita-Salute San Raffaele (Milan, Italy) and Créteil University Eye Clinic (Créteil, France), we enrolled patients 50 years of age and older, visited between January 2017 and January 2019, who were diagnosed with either treatment-naïve pMNV or neovascular AMD. At the time of diagnosis, all patients underwent a comprehensive ophthalmologic evaluation, spectral-domain optical coherence tomography, fluorescein angiography, indocyanine green angiography, and optical coherence tomography angiography. Univariate comparison between pMNV and neovascular AMD groups was performed to identify the main clinical predictors for pMNV. The selected predictors were taken into a binomial logistic regression and eventually served as the basis for the development of InCASEOf scoring system. Receiver operating characteristic (ROC) curves were used to study the model performance. Forty-eight right eyes from 48 patients with pMNV and 39 right eyes from 39 patients with neovascular AMD were considered in this study. Age (+ 2 points), sex (+ 2 points), choroidal thickness (+ 2 points), early pachyvessels (+ 2 points), and evidence of MNV at OCTA (+ 3 points) turned out to be predictors for pMNV. Four additional factors significant at univariate analysis were considered: type 2 and type 3 MNVs and presence of intraretinal fluid (− 0.5 points each), and presence of subretinal fluid (+ 0.5 points). InCASEOf scoring system was built with a high score of 11.5 points. The cutoff value of 6.5 showed good accuracy in separating pMNVs from neovascular AMDs. InCASEOf is a straightforward clinical scoring system, accessible to comprehensive ophthalmologists, with the purpose of enabling easy distinction and expert-like diagnosis of pMNV and neovascular AMD in patients aged 50 years or older.

## Introduction

Pachychoroid disease and age-related macular degeneration (AMD) are two important causes of exudative maculopathy. Although they are two distinct entities, developing along different pathogenetic pathways, they share common mechanisms, such as retinal pigment epithelium (RPE) degeneration and choroidal dysfunction, which may eventually stimulate the development of macular neovascularization (MNV) in both diseases^1,2^.

The term pachychoroid, firstly introduced into the literature in 2013 by Warrow et al.^3^, defines a peculiar phenotype characterized by specific changes to the choroidal anatomy, that is focal or diffuse dilation of Haller’s layer vessels (“pachyvessels”) paralleled with choriocapillaris and Sattler’s layer attenuation, choroidal hyperpermeability on indocyanine green angiography (ICGA), with or without RPE abnormalities overlying the pachyvessels^4,5^. The pachychoroid phenotype predisposes to a spectrum of diseases including pachychoroid pigment epitheliopathy, central serous chorioretinopathy (CSC), pachychoroid neovasculopathy (PNV), polypoidal choroidal vasculopathy/aneurysmal type 1 neovascularization, focal choroidal excavation, peripapillary pachychoroid syndrome, and pachychoroid geographic atrophy^3,5–10^. Noteworthily, the occurrence or exudation of MNV may complicate all pachychoroid disorders (pMNV)^6,9–12^.

Exudative neovascular AMD represents the leading cause of permanent central vision loss among subjects older than 50 years. In neovascular AMD, exudation may develop from pathologic type 1 (sub-RPE), type 2 (sub-retinal), or type 3 (intra-retinal) MNV^13^.

The pathogenesis of pMNV development in pachychoroid disorders is still unclear and may be different from drusen-related mechanisms involved in neovascular AMD. It is still hard to state if choriocapillaris attenuation is primarily due to the mechanical compression by outer choroidal pachyvessels or if it represents the primary event causing secondary passive overflow into the large choroidal veins with their chronic dilation^1,14–17^. Whatever the primum movens of pachychoroid changes is, the choriocapillaris obliteration and atrophy along with RPE damage and degeneration are recognized as the pivotal events contributing to the creation of an ischemic milieu and triggering the neovascular process through the release of angiogenic factors^1,14–16^. Although patients affected by pMNV are usually younger and show thicker choroids compared to neovascular AMD patients, pMNVs share many clinical manifestations in common with neovascular AMD^18^.

We still lack consolidated consensus on the diagnosis of pachychoroid disease^19^ and clear definitions allowing to differentiate pMNV from neovascular AMD have not yet been satisfactorily established. Several authors^1,2,11,16,18,20^ have warned against the misdiagnosis of pMNV as neovascular AMD and have investigated the differences between these two entities with respect to clinical characteristics, genotype distribution, and anti-vascular endothelial growth factor (VEGF) response. In a retrospective analysis, Miyake et al.^2^ found that at least 20% of eyes diagnosed as having AMD before the advent of the “pachychoroid era”, should have been diagnosed as pMNVs instead. Similarly, in a recent study, Borrelli et al.^12^ reported that the pMNV prevalence in their study cohort was 25.2%. Most interestingly, 15.4% of pMNV patients had been misdiagnosed as AMD patients.

Our study aimed to identify the predictive factors for pMNV diagnosis in patients aged 50 years and older affected by pMNV, and to create a novel scoring system for case detection and easy distinction between pMNV and neovascular AMD in non-specialized ophthalmology settings. A correct diagnosis would allow a more effective and customized management of these patients in real-life practice.

## Methods

This study was a retrospective cross-sectional analysis. The Institutional Review Board (IRB) of Creteil University Eye Clinic and University Vita-Salute San Raffaele approved the study and informed consent was obtained from all subjects of both centers, in agreement with the Declaration of Helsinki for research involving human subjects.

We included patients (1) who visited the “Macula Service” of Créteil University Eye Clinic (Créteil, France) and the “Medical Retina and Imaging Unit” of University Vita-Salute San Raffaele (Milan, Italy) between January 2017 and January 2019, (2) who were diagnosed with either treatment-naïve pMNV or neovascular AMD, and (3) who were older than 50 years. The medical records of these patients were collected and retrospectively analyzed between September 10, 2019 and September 10, 2020.

Patients with any of the following conditions were excluded: (1) MNV secondary to high myopia, trauma, angioid streaks, uveitis, or any other exudative maculopathy not including AMD and pachychoroid disease; (2) history of anti-VEGF injections, verteporfin photodynamic therapy (vPDT), and ocular surgery other than for cataract (3) history or evidence of other retinal and optic nerve disorders.

At the time of diagnosis, all patients underwent comprehensive ophthalmologic evaluation including best-corrected visual acuity (BCVA) assessment, slit-lamp biomicroscopy and fundus examination by an experienced retina specialist, infrared reflectance (IR), fundus autofluorescence (FAF), spectral-domain optical coherence tomography (OCT), fluorescein angiography (FA), ICGA and optical coherence tomography angiography (OCTA). IR, FAF, SD-OCT, FA, and ICGA images were acquired using Spectralis HRA + OCT (Heidelberg Engineering, Heidelberg, Germany). Horizontal line scans through the fovea center were obtained using the 1024 × 49 dense raster scanning protocol, with a field of view of 30° × 30°. Enhanced-depth imaging (EDI) OCT images were routinely obtained in all patients, to measure the sub-foveal choroid thickness. All patients underwent OCTA imaging using SD-OCTA AngioVue XR Avanti (Optovue Inc., Fremont, California, USA) and/or SS-OCTA PLEX Elite 9000 devices (Carl Zeiss Meditec Inc., Dublin, CA, USA). For each eye in the study, OCTA images were acquired using the 3 × 3 mm and 6 × 6 mm scan patterns, centered on the fovea.

Definition of Pachychoroid-associated MNV and Neovascular AMD.

In this study, pMNV was diagnosed if all the following criteria were met, as previously reported^2,12^: (1) MNV in at least one eye; (2) subfoveal choroidal thickness ≥ 200 µm in both eyes; (3) no evidence of drusen in both eyes (presence of nonextensive [total area, 125 µm circle] hard drusen [< 63 µm] [Age-Related Eye Disease Study level 1, no AMD] or either pachydrusen as defined by Spaide et al.^21^ was instead allowed); (4) history of CSC or presence of one of the imaging characteristics among presence of choroidal vascular hyperpermeability, RPE abnormalities consistent with pachychoroid disease, and attenuation/thinning of the choriocapillaris and Sattler vessels overlying dilated outer choroidal vessels (pachyvessels) below the MNV lesion.

Neovascular AMD was diagnosed if the following criteria were met, as previously reported^2,12^: (1) patients with MNV and other findings corresponding to Age-Related Eye Disease Study levels 2, 3, and 4 (extensive hard drusen, soft drusen [intermediate, ≥ 63 and < 125 µm; large, ≥ 125 µm], reticular pseudodrusen, focal hyperpigmentation, or RPE and outer retinal atrophy), (2) subfoveal choroidal thickness < 200 µm in at least one eye, or no CSC/pachychoroid pigment epitheliopathy characteristics.

### Primary outcome measure

The study primary outcome was the occurrence of MNV in the context of either pachychoroid disease or AMD during the observational period. The diagnostic labels recorded on the Heidelberg OCT database from both study centers (i.e. “PNV”, “CNV in pachychoroid”, “MNV in pachychoroid”, “MNV in CSC”, “CNV in CSC”, “neovascular AMD”, “CNV in AMD”, and “MNV in AMD”) were used to detect the patients of interest and matched with the diagnosis reported on the clinical records of the patients. Structural OCT and dye angiography (FA and ICGA) images were further reviewed by two independent retina specialists from both centers (P.A. and G.M.C. at Creteil University Eye Clinic, and E.B. and R.S. at University Vita-Salute San Raffaele) to confirm the primary diagnosis.

The readers established the diagnosis in an independent and blinded fashion, according to the criteria mentioned above (see Definition of Pachychoroid-associated MNV and Neovascular AMD). Thereafter, they met to compare the level of agreement, and disagreements were resolved by further discussion and open adjudication to yield a single assessment for each case. In those cases in which the two graders did not agree on a single consensus result, the final decision was made by a third expert in retinal disorders (V.C. and G.Q. at Creteil University Eye Clinic and University Vita-Salute San Raffaele, respectively).

### Covariates

Covariates were selected on the basis of the main demographical characteristics and clinical findings associated with the pachychoroid phenotype in the most recent literature and included: age ≤ 65 years, male sex, choroidal thickness > 300 µm, presence of flat irregular pigment epithelium detachment (FIPED), MNV type, presence of polypoidal choroidal vasculopathy, sub-retinal hyperreflective exudation/material, sub-retinal fluid (SRF), intra-retinal fluid, leaking points, early pachyvessels, late hyperfluorescence, late ICGA plaque, gravitational tracks, MNV evidence at OCTA.

### Statistical analysis

All data were analyzed using SPSS software (IBM SPSS Statistics 26.0, Ontario, Canada). Shapiro–Wilk test was used to determine assimilability to normal distribution.

Univariate comparison between the two groups’ demographic characteristics was performed using two-tailed Student’s t-test for independent groups and χ^2^ test for quantitative and qualitative variables, respectively.

For the analysis of the selected covariates (see above), continuous data (age and choroidal thickness) were transformed into categorical variables. Odds ratio calculation was performed to identify the predictors of MNV secondary to pachychoroid and univariate comparison between the two groups was performed using the χ^2^ test for categorical data or Fishers Exact Test. Categorical variables shown to be significant with a Bonferroni corrected *p* < 0.01 by univariate analysis were subsequently taken into a binomial logistic regression model. Factors with *p* < 0.05 in the adjusted analysis were considered statistically significant and incorporated into the risk prediction model.

The B coefficient of all significant factors, namely the parameter estimate predicting the log odds (logit) of the dependent variable, was considered as a whole number, disregarding the decimal places, as previously reported^22^. The numbers so obtained for all factors were assumed as the points contributing to the final simplified predictive score. The factors resulted significant at univariate analysis but non-significant at logistic regression were integrated into the scoring system and given a minus or plus half-point (depending on the sign of their B coefficient at multivariate analysis).

Receiver operating characteristic (ROC) curves were used to study the model performance by evaluating the area under the curve (AUC), with an AUC of 0.5 indicating no discrimination ability and an AUC of 1.0 indicating maximal discrimination ability. The optimal cutoff point for pMNV diagnosis was estimated as the value that warranted the best combination of true positive rate (sensitivity) and false positive rate (1-specificity).

### Abstract presentation

The abstract of this research has been presented as Free Paper at EURETINA Virtual Congress 2021.

## Results

Forty-eight right eyes from 48 patients with MNV secondary to pachychoroid and 39 right eyes from 39 patients with neovascular AMD were extracted from the electronic database of Creteil University Eye Clinic and University Vita-Salute San Raffaele and considered in this study. All patients were Caucasian and the baseline demographical characteristics of enrolled patients are summarized in Table 1. A statistically significant difference between the two study groups was detected in terms of age, sex, SFCT and vPDT treatment.

**Table 1:** Demographics of the study population at the time of the first diagnosis.

Mean ± SD (CI), no. (%)			
Characteristic	pMNV (N = 48)	nAMD (N = 39)	*P* value
Age	65.35 ± 10.99 (61.25–67.46)	76.79 ± 8.08 (74.26–79.33)	< 0.001
Sex, male	33 (68.75)	13 (33.33)	0.001
SFCT (μm)	360.65 ± 104.16 (331.18–390.11)	225.05 ± 93.44 (195.72–254.38)	< 0.001
vPDT treatment^a^	18 (37.50)	1 (2.56)	< 0.001

CI = Confidence Intervals; IVT = Intravitreal injection; MNV = Macular neovascularization; nAMD = Neovascular age-related macular degeneration; SD = Standard Deviation; SFCT = Subfoveal choroidal thickness; vPDT = Verteporfin Photodynamic therapy; pMNV = Pachychoroid disease complicated by MNV.

^a^Number of patients undergone vPDT after the first diagnosis of MNV.

Among the investigated factors, age, sex, choroidal thickness, MNV type, sub-retinal fluid, intra-retinal fluid, early pachyvessels, and evidence of MNV at OCTA were shown to be significant risk factors for pMNV by univariate analysis (Table 2).

**Table 2:** Results of the univariate analysis.

Variable	No. (%)				
	pMNV (N = 48)		nAMD (N = 39)	OR	*p* value
Age ≤ 65	25 (52.08)		3 (7.69)	13.04	**< 0.001**
Sex, male	33 (68.75)		13 (33.33)	4.40	**0.001**
SFCT > 300	33 (68.75)		8 (20.51)	8.53	**< 0.001**
Drusen^a^	18 (37.50)		24 (61.54)	0.38	0.03
FIPED	33 (68.75)		24 (61.54)	1.38	0.48
**MNV type**					
Type 1	40 (83.33)		25 (64.10)		**0.006**
Type 2	2 (4.17)		2 (5.13)		
Mixed	6 (12.50)		4 (10.26)		
Type 3	0		8 (20.51)		
PCV	2 (4.17)		4 (10.26)	0.38	0.21
SRF	46 (95.83)		28 (71.80)	9.04	**0.006**
IF	6 (12.50)		15 (38.46)	0.23	**0.007**
SHM	21 (43.75)		16 (41.03)	1.12	0.80
Gravitational tracks	11 (28.30)		4 (10.26)	2.60	0.13
Leaking points	7 (14.58)		0	1.17	0.04
Early pachyvessels	36 (75.00)		17 (43.59)	3.88	**0.004**
Late hyperpermeability	33 (62.26)		26 (66.67)	1.02	0.97
Hypercyanescent plaque	22 (45.83)		17 (43.59)	1.70	0.25
MNV evidence at OCTA	45 (93.75)		27 (69.23)	6.67	**0.006**

IF = Intraretinal fluid; OR = Odds Ratio; FIPED = Flat irregular pigment epithelium detachment; MNV = Macular neovascularization; nAMD = Neovascular age-related macular degeneration; OCTA = Optical Coherence Tomography Angiography; OR = Odds ratio; PCV = Polypoidal choroidal vasculopathy; pMNV = Pachychoroid disease complicated by MNV; SFCT = Subfoveal choroidal thickness; SHM = Sub-retinal hyperreflective Material; SRF = Sub-retinal fluid.

^a^More than 6 drusen (> 63 μm each) in ETDRS grid. Significant values are in bold.

**Figure 1:**
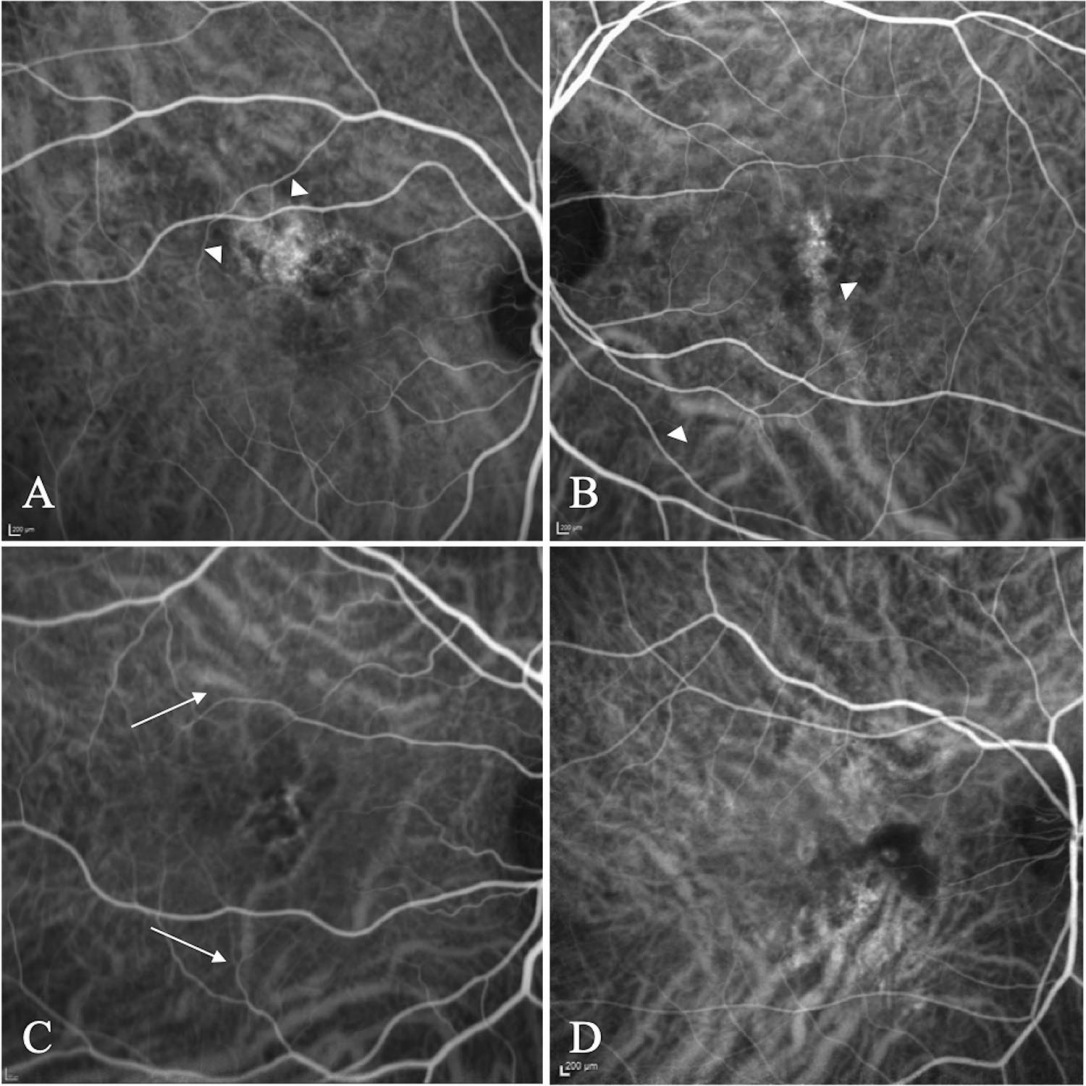
Exemplar early-phase ICGA images showing posterior pole pachyvessels in patients diagnosed with MNV secondary to pachychoroid disease. Abnormally dilated choroidal vessels can be preferentially localized in a half of the macula (**A** superior intervortex veins are dominant compared to the inferior ones, **B** inferior vortex veins are dominant compared to the superior ones) or diffusely distributed over the whole macula (**C** spaced pattern, **D** dense pattern). The normal horizontal watershed zone has disappeared showing instead collateral veins due to anastomoses between the superior and inferior vortex veins. The pachyvessels typically do not taper towards the macula and display abrupt, club-shaped posterior termination.

**Figure 2:**
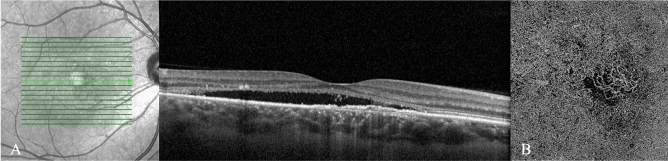
(**A**) Enhanced-depth imaging optical coherence tomography reveals dilated Haller’s layer vessels and attenuation of the choriocapillaris, and a flat irregular pigment epithelium detachment (FIPED) associated with subretinal fluid (SRF) and subretinal hyperreflective foci. (**B**) The enface OCT angiography demonstrates a neovascular lesion with tangled filamentous pattern within the FIPED.

Binomial logistic regression was performed to ascertain the effects of these factors on the likelihood that participants have pMNV rather than neovascular MNV (Table 3). The logistic regression model resulted statistically significant (χ^2^ = 71.65, *p* < 0.001). The B coefficient of each significant predictive factor was considered as a whole number, disregarding the decimal places. The numbers so obtained for all factors were assumed as the points contributing to the final simplified predictive score. Age (+ 2 points), sex (+ 2 points), choroidal thickness (+ 2 points), early pachyvessels (+ 2 points, Fig. 1), and evidence of MNV at OCTA (+ 3 points, Fig. 2) were factors associated with an increased likelihood of exhibiting pMNV. Four extra factors significant at univariate analysis but non-significant at logistic regression were integrated in the scoring system. Most specifically, type 2 and type 3 MNVs, and the presence of IF were assumed to give − 0.5 points each, whereas the presence of SRF was assumed to give + 0.5 points. InCASEOf scoring system was built based on these results with a high score of 11.5 points (Table 4).

**Table 3:** Results of logistic regression analysis.

	B	S.E	Wald	df	Sign	Exp (B)
Male sex	2.253	0.860	6.856	1	**0.009**	9.513
Age ≤ 65	2.465	0.966	6.517	1	**0.011**	11.767
SFCT > 300	2.172	0.901	5.818	1	**0.016**	8.779
CNV type			0.155	3	0.984	
Type 1 MNV	− 0.461	1.172	0.155	1	0.694	0.630
Type 2 MNV	− 22.181	21,861.790	0.000	1	0.999	0.000
Type 3 MNV	− 20.604	11,866.749	0.000	1	0.999	0.000
SRF	0.332	1.247	0.071	1	0.790	1.393
IF	− 1.905	1.136	2.811	1	0.094	0.149
Early pachyvessels	2.554	0.943	7.328	1	**0.007**	12.853
Octa MNV evidence	3.187	1.289	6.113	1	**0.013**	24.208

Significant values are in bold.

**Table 4:** InCASEOf scoring system.

	Predictors	B coefficient	Score
In	I type MNV:		
	Type II MNV		− 0.5
	Type III MNV		− 0.5
C	Choroidal thickness > 300 μm	2	+ 2.0
A	Age ≤ 65 years	2	+ 2.0
S	Sex M	2	+ 2.0
E	Early pachyvessels	2	+ 2.0
O	OCTA MNV evidence	3	+ 3.0
f	Fluid		
	Intra-retinal		− 0.5
	Sub-retinal		+ 0.5

**Figure 3:**
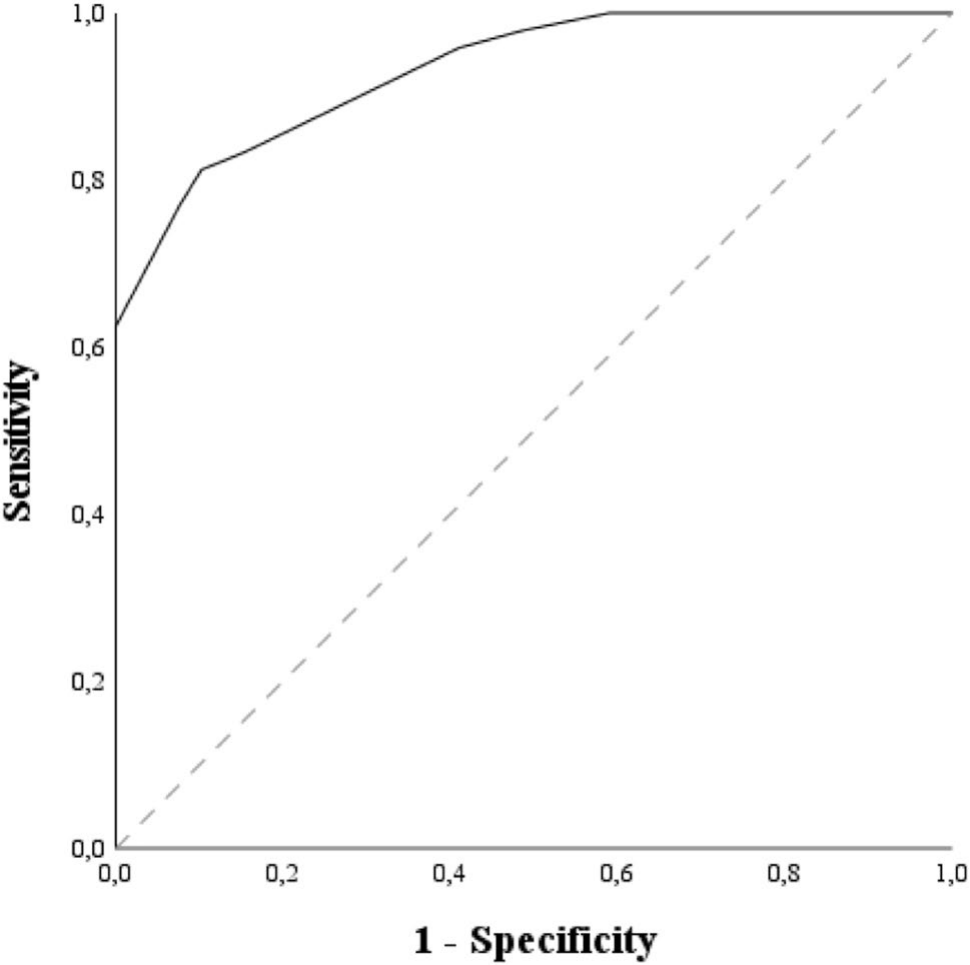
Receiver Operating Characteristic (ROC) Curve and Area Under the Curve (AUC) calculated for InCASEOf scoring system (solid line; AUC = 0.93). The gray dashed diagonal line serves as an imaginary reference line representing a non-discriminatory test.

ROC curves were calculated for InCASEOf scoring system (Fig. 3). The AUC was 0.93 (standard error [SE], 0.02; asymptotic *p* < 0.001; confidence limits, 0.88–0.98). The cutoff value of 6.5 (sensitivity: 0.81, specificity: 0.90) was able to separate pMNVs from neovascular AMDs.

**Figure 4:**
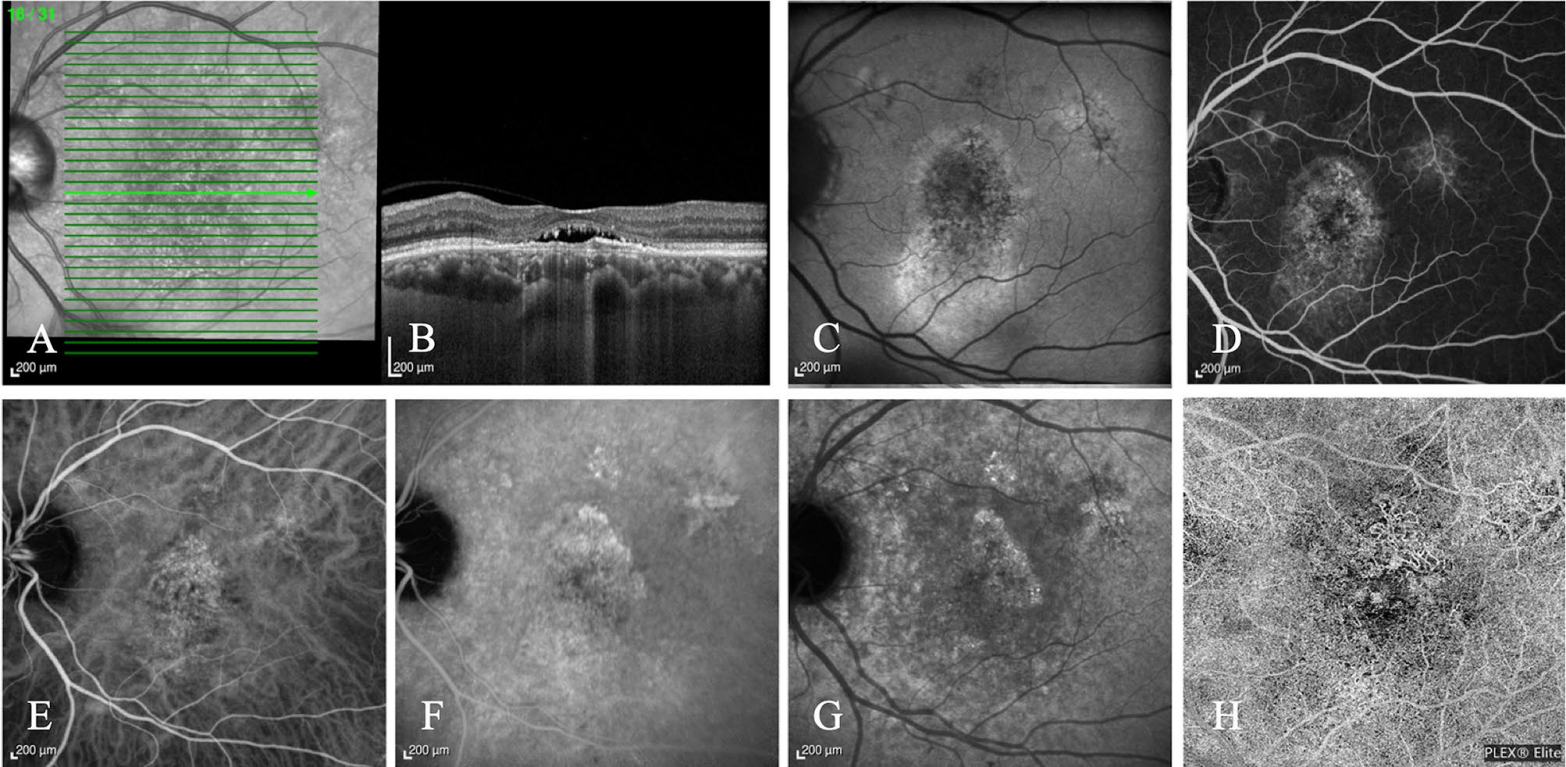
Multimodal imaging of a 67-year-old Caucasian male diagnosed with pMNV. (**A**,**B**) Enhanced-depth imaging optical coherence tomography shows an increased subfoveal choroidal thickness (383 µm) with dilated Haller’s layer veins and thinning of the choriocapillaris, and a flat irregular pigment epithelium detachment (FIPED) associated with subretinal fluid and subretinal hyperreflective foci. (**C**) Fundus autofluorescence reveals a hyperautofluorescent gravitational track. (**D**) Fluorescein angiography (FA) shows an early pinpoint hyperfluorescence. (**E**) Macular pachyvessels are visible at early phase of indocyanine green angiography. A bigger hyperfluorescent plaque and two smaller satellite plaques consistent with multifocal type 1 macular neovascularization are evident at (**F**) intermediate and (**G**) late phases of ICGA. (**H**) Flow within a neovascular lesion corresponding to the FIPED is seen on the enface OCT angiography image. I = 0 (type 1 MNV); C = + 2 (> 300 μm); A = 0 (> 65 years); S = + 2 (male); E = + 2 (visible early pachyvessels); O = + 3 (evidence of MNV at OCTA); f = + 0.5 (presence of SRF). The patient scores 9.5.

**Figure 5:**
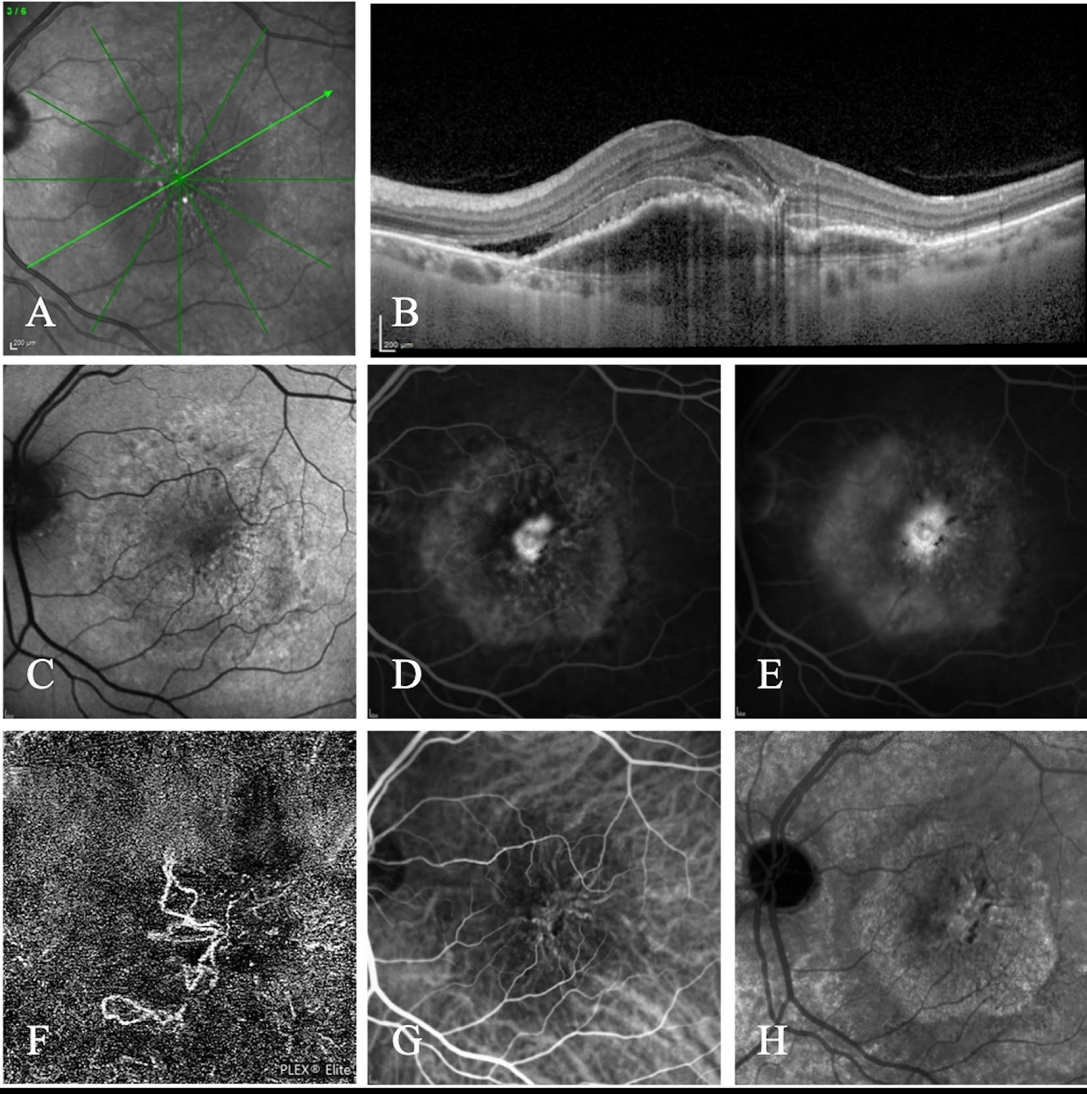
Multimodal imaging of a Caucasian 83-year-old female diagnosed with mixed MNV secondary to neovascular AMD. (**A**,**B**) The OCT scan reveals a fibrovascular pigment epithelium detachment associated with sub-retinal fluid, sub-retinal hyperreflective material and hyperreflective foci. The sub-foveal choroidal thickness was 216 μm. (**C**) The fundus autofluorescence shows a macular granular hyper/hypoautofluorescence. The fluorescein early (**D**) and late (**E**) angiograms display a homogeneous well-defined intense central hyperfluorescence (type 2 MNV) and a pinpoint surrounding hyperfluorescence (type 1 MNV) with late leakage of both components. (**F**) OCTA shows the presence of flow within the neovascular lesion with irregular filamentous pattern. (**G**) The early indocyanine green angiogram clearly reveals a central net of small vessels (type 2 MNV). (**H**) The late phase angiogram demonstrates a hyperfluorescent plaque (type 1 MNV). I = 0 (mixed MNV); C = 0 (< 300 μm); A = 0 (> 65 years); S = 0 (female); E = 0 (No early pachyvessels); O = 3 (evidence of MNV at OCTA); f = + 0.5 (presence of SRF). The patient scores 3.5.

Figures 4 and 5 show two examples of InCASEOf scoring system application.

## Discussion

In this retrospective analysis, we attempted to identify the main clinical predictors for pMNV in Caucasian patients older than 50 years. The selected predictors further served as the basis for the development of a novel scoring system, dubbed InCASEOf scoring system, aiming at guiding the differential diagnosis between pMNV and neovascular AMD in non-specialized ophthalmologic settings. The idea to provide a user-friendly scoring system as a tool to correctly detect pMNVs stemmed from the results of a recent retrospective analysis^12^, showing that 15.4% of the patients managed in a retina referral center had been misdiagnosed as having neovascular AMD while affected by pMNV.

We identified 5 main predictors of pMNV: MNV evidence at OCTA, early pachyvessels, choroidal thickness > 300 μm, age ≤ 65 years, and male sex. Further, we added 4 additional half-point factors which showed statistical significance at univariate analysis but not at multivariate analysis: type 2 and type 3 MNVs and the presence of IF were assumed to give − 0.5 each, whereas the presence of SRF was assumed to give + 0.5. Finally, we found that a score ≥ 6.5 provided good accuracy in discriminating pMNV from neovascular AMD cases.

The most robust predictor was the evidence of MNV at OCTA analysis in patients with pMNV. Different studies have already demonstrated that OCTA can detect MNVs more frequently than the other imaging modalities in patients with chronic central serous corioretinopathy^14,23^. OCTA has shown a good diagnostic accuracy also for MNVs secondary neovascular AMD^24^. However, in our study the rate of MNV detection by OCTA was 93.75% in pMNV group and 69.23% in neovascular AMD group (*p* < 0.006).

Dilated choroidal veins (pachyvessels) and increased choroidal thickness are the hallmarks of pachychoroid-related disorders^1,25^. Earlier studies defined “pachychoroid” as choroidal thickness > 270 μm, paralleled with the presence of pachyvessels^6^. With respect to pMNV, Miyake et al.^2^ found a mean sub-foveal choroidal thickness (SFCT) of 310 ± 53 μm for pMNV population compared to 208 ± 100 for neovascular AMD population. Another study^12^ reported a mean SFCT of 387.6 ± 81.9 and 152.5 ± 74.4 for pMNV and neovascular AMD, respectively. Recently, some authors^19^ have highlighted the inaccuracy of the SFCT as a diagnostic criterion for pachychoroid disorders and have deflected the attention from choroidal thickness to the choroidal circulation itself. Intriguingly, several recent studies^26–30^ have postulated that choroidal vortex vein congestion and subsequent formation of intervortex venous anastomoses might have an integral role in the pathogenesis of pachychoroid. In particular, Matsumoto and colleagues^26^ demonstrated the remodeling of choroidal drainage routes by the development of anastomotic connections between superotemporal and inferotemporal vortex vein systems at the posterior pole in eyes with PNV. Spaide et al.^27^ introduced the concept of venous overload choroidopathy to explain the vascular abnormalities shared by all the pachychoroid disorders. We found that both the presence of early ICGA pachyvessels crossing over the macula and SFCT > 300 μm and were predictors of pMNV.

Two studies recently investigated type 1 MNV incidence and associated factors in eyes with CSC. Whilst Shiragami et al.^25^ demonstrated that chronic CSC, female sex, and poor BCVA were associated with pMNV, Savastano et al.^31^ found that younger female subjects with greater choroidal thickness and BCVA preservation had a lower occurrence of pMNV. Our results showed that both male sex and age ≤ 65 years can be considered predictive factors for PNV.

Finally, although type 2 and type 3 MNV, IF, and SRF did not result significative in the regression model, we decided to integrate them into the scoring system because they turned to be protective/predictive factors for pMNV at univariate analysis, consistently with the existing literature^1,6,11,14,23,25,32^.

Considering its simplicity, InCASEOf scoring system would most appropriately be used within non-specialized ophthalmological settings for differentiating between pMNVs and neovascular AMDs.

Yanagi et al.^1^ proposed a new classification categorizing MNVs into 4 groups, based on the presence or absence of drusen and pachychoroid features. This classification, however, still does not address the issue of how to best identify the main distinctive pachychoroid features in the practice.

Hosada et al.^33^ recently applied an unsupervised machine learning algorithm to classify MNV patients into two groups, i.e. AMD-type and PNV-type, and eventually created a scoring system based on 7 parameters, namely, age, sex, central retinal thickness and SFCT in the MNV-affected eye, SFCT in the fellow eye, the presence of choroidal vascular hyperpermeability in either eye, and the presence of soft drusen. The authors found that 46.2% of MNV patients belonged to pachychoroid spectrum, which was higher than the proportion described in their previous reports^2^. Nonetheless, this scoring system was based on only Japanese participants.

A correct and prompt diagnosis of pMNV or neovascular AMD is crucial to provide patients with more effective and personalized management. Indeed, although treatment models and therapeutic outcomes are beyond the scope of this retrospective analysis, it should be borne in mind that the different responses of MNVs to treatment may depend on their distinct etiologies^14^. The role of VEGF has been strongly advocated for the progression of pMNV^15,20,34^, as well as in neovascular AMD. However, Hata et al.^16^ demonstrated that the VEGF levels in PNV are significantly lower when compared to neovascular AMD, suggesting that VEGF drives the neoangiogenetic process differently in the two diseases. For this reason, many cases of pMNV might be misinterpreted in real-life practice as being unresponsive neovascular AMD while they should just be managed differently by other/further therapeutic strategies. In this regard, a previous study by Lee et al.^35^ demonstrated excellent efficacy of adjuvant vPDT combined with intravitreal anti-VEGF treatment in the control of the exudative changes and improvement of vision in eyes with type 1 neovascularization associated and thickened choroid.

InCASEOf scoring system may facilitate general ophthalmologists in the identification of pMNV patients for which combined vPDT and anti-VEGF therapy can be decisive.

Two limitations of this research are the relatively small sample size and the lack of ethnic heterogeneity, as our study population only included Caucasian individuals. However, to the best of our knowledge, this is the first study conducted on a Caucasian population to develop and propose a straightforward clinical scoring system with the purpose of enabling expert-like diagnosis of pMNV and neovascular AMD.

In conclusion, in the present study, we investigated the differences between pMNV and neovascular AMD by analyzing the demographical characteristics and clinical features observed in our two study groups. We further used the predictors detected through the statistical analysis for the elaboration of a simple scoring system accessible to comprehensive ophthalmologists for an easier differential diagnosis between pMNV and neovascular AMD in subjects aged 50 years or older, and for a more effective and customized management and treatment of these patients.
